# The LEAP checklist for laboratory evaluation and analytical performance characteristics reporting of clinical measurement procedures

**DOI:** 10.11613/BM.2023.030505

**Published:** 2023-10-15

**Authors:** Tze Ping Loh, Brian R Cooke, Thi Chi Mai Tran, Corey Markus, Rosita Zakaria, Chung Shun Ho, Elvar Theodorsson, Ronda F Greaves

**Affiliations:** 1 Department of Laboratory Medicine, National University Hospital, Singapore, Singapore; 2 Department of Clinical Biochemistry, PathWest Laboratory Medicine, Fiona Stanley Hospital, Murdoch, Australia; 3 Faculty of Medical Technology, Hanoi Medical University, Hanoi, Vietnam; 4 Department of Clinical Biochemistry, National Children’s Hospital, Hanoi, Vietnam; 5 Flinders University International Centre for Point-of-Care Testing, Flinders Health and Medical Research Institute, Adelaide, Australia; 6 RMIT University, School of Health and Biomedical Sciences, Victoria, Australia; 7 Murdoch Children’s Research Institute, Melbourne, Australia; 8 Department of Chemical Pathology, The Chinese University of Hong Kong, Prince of Wales Hospital, Shatin, Hong Kong; 9 Department of Biomedical and Clinical Sciences, Division of Clinical Chemistry and Pharmacology, Linkoping University, SE-58183 Linkoping, Sweden; 10 Department of Paediatrics, The University of Melbourne, Melbourne, Australia; 11 Victorian Clinical Genetics Services, Murdoch Children’s Research Institute, Parkville, Australia

**Keywords:** emerging technology(ies), method verification, method validation

## Abstract

Reporting a measurement procedure and its analytical performance following method evaluation in a peer-reviewed journal is an important means for clinical laboratory practitioners to share their findings. It also represents an important source of evidence base to help others make informed decisions about their practice. At present, there are significant variations in the information reported in laboratory medicine journal publications describing the analytical performance of measurement procedures. These variations also challenge authors, readers, reviewers, and editors in deciding the quality of a submitted manuscript. The International Federation of Clinical Chemistry and Laboratory Medicine Working Group on Method Evaluation Protocols (IFCC WG-MEP) developed a checklist and recommends its adoption to enable a consistent approach to reporting method evaluation and analytical performance characteristics of measurement procedures in laboratory medicine journals. It is envisioned that the Laboratory Evaluation and Analytical Performance Characteristics (LEAP) checklist will improve the standardisation of journal publications describing method evaluation and analytical performance characteristics, improving the quality of the evidence base that is relied upon by practitioners.

## Introduction

The reporting of a measurement procedure and its analytical performance following method evaluation in a peer-reviewed journal is an important means for clinical laboratory practitioners to share their findings. It represents an important source of evidence base to help others make informed decisions about their practice. These publications must report the essential components of method evaluation and their analytical performance characteristics in a standardised, consistent manner to enable replication and to improve the generalisability of the findings (*1*). This will also facilitate the pooling of findings from individual studies *e.g.* for meta-analysis. At present, there are significant variations in the information reported in laboratory medicine journal publications describing the analytical performance of measurement procedures (*2*). These variations also challenge authors, readers, reviewers, and editors in deciding the quality of a submitted manuscript.

The International Federation of Clinical Chemistry and Laboratory Medicine Working Group on Method Evaluation Protocol (IFCC WG-MEP) aimed to develop a checklist and recommends its adoption to enable a consistent approach to reporting method evaluation and analytical performance characteristics of measurement procedures in laboratory medicine journals.

## Methods

### Checklist development

A draft checklist was developed by the IFCC WG-MEP following the recommendations and toolkit of the Enhancing the Quality and Transparency Of health Research (EQUATOR) Network (*3*). This draft was presented to the full WG-MEP, including corresponding members, at the annual meeting held during the IFCC WorldLab conference in Rome on 21^st^ May 2023, and suggestions for improvements were incorporated into the submitted version. After extensive discussion and consensus agreement of the working group members, the checklist was finalised for multi-journal publication as an open-access offering to allow for free dissemination and use by clinical laboratories, manufacturers, other related journals, editors, reviewers, readers, and authors.

## Results

The Laboratory Evaluation and Analytical Performance Characteristics (LEAP) checklist is presented in Table 1. This table encompasses various main elements and requirements of method evaluation for clinical testing that should be included in a published paper. Authors are advised to adequately address and provide evidence for each item in the checklist to ensure that all necessary issues of method evaluation are fully addressed. Authors need to determine if the study involves method validation (*e.g.* when describing an emerging technology, a new measurement procedure, or a laboratory-developed test) or method verification (*e.g.* when evaluating an established, regulatory-approved commercial measurement procedure) and report the components accordingly. In addition, the analytical performance specifications should be defined *a priori* according to the clinical purpose of the measurement procedure. Appropriate statistical tests and quantitative results should be reported and assessed against the *a priori-*defined analytical performance specification to determine if the measurement procedure fits the intended clinical use.

**Table 1:** Laboratory Evaluation and Analytical Performance Characteristics (LEAP) checklist

**Item**	**No.**	**Recommendation**
Title	1	Indicate whether the study involves a. Method verification of an established commercial measurement procedure, or b. Method validation of a modified/ novel measurement procedure or a laboratory-developed test.
Abstract	2	a. Indicate the key performance characteristics studied. b. Provide numerical absolute and relative results of performance characteristics such as imprecision, bias, and linearity instead of qualitative statements.
Introduction	3	a. For novel technology or measurement procedure, indicate the clinical need it is addressing and the clinical pathway within which it is applied (*5*, *6*). b. For existing/commercial technology or measurement procedure, indicate the intended clinical context (*e.g.,* clinical condition, population, clinical pathway) within which the technology or measurement procedure will be applied.
Ethics	4	a. If patient samples or data are being used in the study, indicate whether ethics approval has been sought, or if appropriate, indicate the reason for the waiver. Compliance with the WMA Declaration of Helsinki should be indicated, where relevant (*7*).
Technology/ measurement procedure	5	a. Describe the technology and/ or measurement procedure used to produce the laboratory results in sufficient detail (*i.e.* including hardware, calibrator/reagent, procedure/protocol, consumables, and software) to allow independent replication of the results. b. Describe the matrix of the material used and, where relevant, the purity of the materials (*e.g.* solvent and standards) used. b. Detail the traceability hierarchy of the higher order reference materials used and its measurement uncertainty if such information is available. c. Indicate whether the technology or measurement procedure has received regulatory approval for clinical use, or whether it is limited for research-use only.
Materials used	6	a. Describe the material used for each analytical performance component in the study (*e.g.,* patient sample, quality control material, external quality assurance material or commercial material), the sample matrix, and if known, the commutability and traceability of the material (demonstrated or otherwise). b. Describe the concentration of the materials used and provide clinical justification for their selection. c. Describe any alteration (*e.g.* dilution, spiking of material) of the sample, where relevant. d. Describe the stability and storage conditions of the material if relevant.
Experimental designs	7	a. The components of analytical performance evaluation include repeatability and reproducibility imprecision, bias, linearity, analytical measurement interval, clinically reportable interval, dilution factor, limits of quantitation, interference study, method comparison, carryover and stability. Noting the components of the method evaluation varies depending on whether validation or only verification is required. b. Describe the number of replicates, runs and days (particularly for precision studies) over which the evaluation was performed. c. Describe the experimental procedures, including storage conditions and sample preparation, used for each evaluation component.
Analytical performance specification	8	a. Define *a priori* analytical performance specifications (*i.e.* acceptance/rejection criteria) for each of the evaluation components with a clear rationale following the Milan consensus (*8*).
Statistical analysis	9	a. Describe the statistical analysis performed to assess each component of the analytical performance characteristics. b. For statistical analysis involving linear regression, statistical models that are robust regarding heteroscedasticity are preferred. c. Of note, regression characteristics, including slope, intercept, coefficient of coefficient, r, and correlation of determination, R^2^, are not properties of linearity and should not be reported in this context.
Analytical performance characteristics	10	a. Summarise the findings for each evaluation component as stated method section. b. Provide an appropriate numerical summary for the performance characteristics. c. Provide confidence intervals and/or P value if formal statistical testing was performed. d. Use the appropriate significant figures when reporting the data. e. Provide data on proficiency testing performance, especially quantitative data on bias.
Outlier results	11	a. Describe the methods used for detecting outliers, detail number of outliers detected in the study, and whether they were excluded with or without replacement. b. Provide possible reasons for the outlier results that are not due to gross blunders to improve understanding of the measurement procedure.
Interpretation	13	a. Interpret the findings of the evaluation study conservatively in the clinical context where the technology or measurement procedure will be applied. b. Compare the findings of the evaluation study against the *a priori*-defined analytical performance specification and discuss whether it is fit for purpose.
Limitations	14	a. Report and discuss any relevant limitations in the study design that may influence/restrict/bias the findings. b. Discuss any analytical limitations uncovered during the evaluation study.
Generalisability	15	a. Discuss the findings of the study in the context of existing literature (*e.g.,* other studies or incumbent technology/measurement procedures).
WMA - World Medical Association.		

## Discussion

The IFCC WG-MEP has developed and proposed a checklist for using peer-reviewed journals when reporting studies related to method evaluation and analytical performance. The checklist includes essential items on which future studies should be based when publishing their results. This LEAP checklist should be used as a guide for authors, journal editors, and peer reviewers of method evaluation studies to ensure that a study is reported in a comprehensive, transparent, and replicable way.

The Standards for Reporting Diagnostic accuracy studies (STARD) checklist first published in 2003 (revised in 2015) has been widely adopted by peer-reviewed journals reporting diagnostic performances (*4*). It has contributed to improved standardisation when reporting such results and has facilitated the ability to pool data for meta-analysis. The LEAP checklist has been developed with similar intention focusing on method evaluation following the principles of the EQUATOR initiative (*3*).

The checklist is specific to the method evaluation. Of note, the establishment and verification of reference intervals are considered outside of the scope of method evaluation for this checklist. Similarly, clinical performance (*i.e.* clinical sensitivity, clinical specificity, accuracy, *etc.*) is also not considered in this checklist and authors are referred to other relevant checklists, such as the STARD 2015 checklist for this information (*4*). However, regarding method evaluation we consider this checklist to be comprehensive.

In summary, it is envisioned that the LEAP checklist will improve the standardisation of journal publications describing method evaluation and analytical performance characteristics, which will in turn improve the quality of the evidence base that is relied upon by practitioners.
